# 1198. Lyme Disease Post-Exposure Prophylaxis by Single-Dose Doxycycline in Three Healthcare Systems

**DOI:** 10.1093/ofid/ofab466.1390

**Published:** 2021-12-04

**Authors:** Grace E Marx, Anna M Schotthoefer, Brian S Schwartz, Evan Draper, Christina G Rivera, John Zeuli, Erica Scotty, Jonathan S Pollak, Amy M Schwartz, Alyssa Beck, Alison F Hinckley

**Affiliations:** 1 Centers for Disease Control and Prevention, Fort Collins, Colorado; 2 Marshfield Clinic Research Institute, Marshfield, Wisconsin; 3 Johns Hopkins Bloomberg School of Public Health, Baltimore, Maryland; 4 Mayo Clinic, Rochester, Minnesota

## Abstract

**Background:**

Lyme disease, the most common tickborne disease in the United States, may be prevented by taking a single 200-mg dose of oral doxycycline after a high-risk bite from a blacklegged tick. Currently, it is not known how Lyme disease post-exposure prophylaxis (PEP) might vary by region and healthcare system. We identified single-dose doxycycline medication orders in three healthcare systems in states with high incidence of Lyme disease and compared associated patient and provider characteristics.

**Methods:**

Electronic health record data during 2012 – 2016 were obtained from three healthcare systems: Geisinger (Pennsylvania), Marshfield Clinic (Wisconsin), and Mayo Clinic (Minnesota/Wisconsin). Creation of analytic variables and analysis were harmonized across the three sites. Medication orders for single-dose doxycycline ≤200 mg that were accompanied by specific key words or diagnostic codes (e.g., tick bite; Lyme disease prevention) were considered evidence of PEP. Manual chart review was performed from a random subset to evaluate the algorithms used to identify PEP.

**Results:**

Among 2,937,585 patients with at least one medication order or clinical encounter during the study period, 14,102 single-dose doxycycline orders for Lyme disease PEP for 13,172 unique patients were identified. The typical patient receiving PEP was older (mean age 51 – 58 years), male (56 – 59%), and non-Hispanic White (81 – 98%). The annual seasonality of medication orders was bimodal, with peaks occurring during April – July and October – November. The most common encounter setting was an outpatient clinic or urgent care center (80 – 91%); medication orders after patient phone calls in the absence of an in-person visit occurred frequently (14 – 19%) in two health systems. Chart abstractions (n=600) revealed instances of PEP prescribed inappropriately (e.g., bite from a non-blacklegged tick; patient with symptoms of acute Lyme disease).

**Conclusion:**

Lyme disease PEP with a single dose of doxycycline was frequently prescribed in healthcare systems where there is a high incidence of Lyme disease. PEP was most commonly prescribed to non-Hispanic Whites over the age of 50 years. Public health initiatives for tickborne disease prevention should include clinician education on the appropriate use of Lyme disease PEP.

**Disclosures:**

**Anna M. Schotthoefer, PhD**, **HelixBind** (Other Financial or Material Support, salary support) **John Zeuli, PharmD**, **INSMED** (Other Financial or Material Support, honoraria for educational speaking)

